# Human double negative T cells target lung cancer via ligand-dependent mechanisms that can be enhanced by IL-15

**DOI:** 10.1186/s40425-019-0507-2

**Published:** 2019-01-22

**Authors:** Junlin Yao, Dalam Ly, Dzana Dervovic, Linan Fang, Jong Bok Lee, Hyeonjeong Kang, Yu-Hui Wang, Nhu-An Pham, Hongming Pan, Ming-Sound Tsao, Li Zhang

**Affiliations:** 1 Toronto General Hospital Research Institute, University Health Network, Toronto, Ontario Canada; 2 Department of Immunology, University of Toronto, Toronto, Ontario Canada; 3 Princess Margaret Cancer Centre, University Health Network, Toronto, Ontario Canada; 4 Sir Run Run Shaw Hospital, College of Medicine, Zhejiang University, Hangzhou, China; 5 Department of Laboratory Medicine and Pathobiology, University of Toronto, Toronto, Ontario Canada; 6 Present address: Sir Run Run Shaw Hospital, College of Medicine, Zhejiang University, Hangzhou, China; 7 Present address: Department of Systems Biology, Mount Sinai Hospital, Toronto, Ontario Canada; 8 University Health Network, Princess Margaret Cancer Research Tower, 101 College St. Rm 2-807, Toronto, Ontario M5G 1L7 Canada

**Keywords:** Adoptive cellular therapy, Double negative T cells, Lung cancer, IL-15

## Abstract

**Background:**

The advents of novel immunotherapies have revolutionized the treatment of cancer. Adoptive cellular therapies using chimeric antigen receptor T (CAR-T) cells have achieved remarkable clinical responses in B cell leukemia and lymphoma but the effect on solid tumors including lung cancer is limited. Here we present data on the therapeutic potential of allogeneic CD3^+^CD4^−^CD8^−^ double negative T (DNT) cells as a new cellular therapy for the treatment of lung cancer and underlying mechanisms.

**Methods:**

DNTs were enriched and expanded ex vivo from healthy donors and phenotyped by flow cytometry. Functionally, their cytotoxicity was determined against primary and established non-small-cell lung cancer (NSCLC) cell lines in vitro or through in vivo adoptive transfer into xenograft models. Mechanistic analysis was performed using blocking antibodies against various cell surface and soluble markers. Furthermore, the role of IL-15 on DNT function was determined.

**Results:**

We demonstrated that ex vivo expanded DNTs can effectively lyse various human NSCLC cells in vitro and inhibit tumor growth in xenograft models. Expanded DNTs have a cytotoxic phenotype, as they express NKp30, NKG2D, DNAM-1, membrane TRAIL (mTRAIL), perforin and granzyme B, and secrete IFNγ and soluble TRAIL (sTRAIL). DNT-mediated cytotoxicity was dependent on a combination of tumor-expressed ligands for NKG2D, DNAM-1, NKp30 and/or receptors for TRAIL, which differ among different NSCLC cell lines. Furthermore, stimulation of DNTs with IL-15 increased expression of effector molecules on DNTs, their TRAIL production and cytotoxicity against NSCLC in vitro and in vivo.

**Conclusion:**

Healthy donor-derived DNTs can target NSCLC in vitro and in vivo*.* DNTs recognize tumors via innate receptors which can be up-regulated by IL-15. DNTs have the potential to be used as a novel adoptive cell therapy for lung cancer either alone or in combination with IL-15.

**Electronic supplementary material:**

The online version of this article (10.1186/s40425-019-0507-2) contains supplementary material, which is available to authorized users.

## Background

Lung cancer is the leading cause of cancer-related deaths worldwide with less than 15% of patients having a 5-year overall survival. Non-small-cell lung cancer (NSCLC) represents 85% of all lung cancer cases. Most patients present with advanced disease and treatment options are based on histology subtype and molecular pathology [1]. Platinum-based chemotherapy remains the cornerstone of treatment in most patients, but response rates are modest and some patients do not tolerate the adverse side effects [2]. In patients whose tumors harbor mutation or re-arrangement in *EGFR*, *ALK*, or *ROS1* genes, targeted therapy improves survival, but invariably patients experience progression due to development of resistance [3].

Immunotherapy represents an innovative approach for the treatment of NSCLC, with several immune checkpoint inhibitors, tumor cell vaccines and adoptive cellular therapies being investigated [4]. Immune checkpoint inhibitors targeting PD-1/PD-L1 have shown improved efficacy and longer duration of response compared to chemotherapy in a subset of patients whose tumors express PD-L1 [5, 6]. Strategies to immunize patients after complete surgical resection with tumor cell vaccines, including the melanoma-associated antigen-A3 (MAGE-A3) and MUC1 vaccines, have so far failed to improve overall survival in early stage NSCLC patients [7, 8]. Finally, adoptive cell therapies for NSCLC are promising but remain limited in clinical use. Clinical trial data show that adoptive therapy of autologous cytokine-induced killer (CIK) cells is well tolerated, with efficiency over conventional chemotherapy [9–11]. Further, tumor infiltrating lymphocytes and CAR-T cell therapy for solid tumors are still in pre-clinical or early clinical phases [12]. Therefore, continued efforts are needed to explore safer and more effective therapies for NSCLC patients.

Double negative T cells (DNTs) comprise 3–5% of the peripheral blood mature T lymphocyte pool as defined by expression of CD3 in the absence of CD4 and CD8. Previously, we demonstrated that ex vivo expanded allogenic DNTs represent a promising cellular therapy for the treatment of acute myeloid leukemia (AML) [13–15]. In those studies, we have established a protocol which allows for ex vivo expansion of therapeutic numbers and clinical grade DNTs with high purity from healthy donors [14, 16]. We have extensively characterized the “off-the-self” nature of DNTs and demonstrated their safety and efficacy in treating AML in patient-derived xenograft (PDX) models [14]. Whether DNTs can be used to target solid tumors remains unclear. Here, we demonstrate that ex vivo expanded DNTs are cytotoxic towards a large panel of NSCLC cell lines in vitro and can inhibit tumor growth in xenograft models. Stimulation of DNTs with IL-15 further enhances their anti-tumor activities. Furthermore, we show that DNTs utilize various mechanisms to recognize and target lung cancer cells, which are dependent on the expression of ligands on cancer cells.

## Materials

Anti-human antibodies specific for CD3 (clone HIT3a), CD4 (clone OKT4), CD8 (clone HIT8a), CD69 (clone FN50), CD25 (clone PC61), NKG2D (clone 1D11), DNAM-1 (clone 118A), Fas ligand (FasL; clone NOK-1), NKp30 (clone P30–15), NKp44 (clone P44–8), NKp46 (clone 9E2), perforin (clone B-D14), granzyme B (clone GB11), CD112 (clone TX31), CD155 (clone SKII.4), NKG2D (clone 1D11), DNAM-1 (clone 11A8), NKp30 (clone P30–15), FasL (clone NOK-1), NKp44 (clone P44–8), membrane TNF-related apoptosis-inducing ligand (TRAIL; clone RIK-2), killer cell immunoglobulin-like receptors (KIRs) CD158a (clone HP-MA4), CD158b (clone DX27), CD158e (clone DX9), CD94 (clone DX22), anti-HLA A/B/C (clone W6/32), anti-HLA-E (clone 3D12), anti-TCRγδ (clone B1), as well as isotype antibodies mouse IgG1, κ (clone RMG1–1), mouse IgG2α, κ (clone RMG2a-62), mouse IgG2β, κ (clone 27–35) and rat IgG1, γ (clone G0114F7) were purchased from Biolegend. Antibodies specific for TRAIL-R1 (clone 69,036), TRAIL-R2 (clone 71,908), TRAIL-R3 (clone 90,906), TRAIL-R4 (clone 104,918), MIC-A/B (clone 159,207), ULBP-1 (clone 170,818), ULBP-2/5/6 (clone 165,903), ULBP-3 (clone 166,510) and ULBP-4 (clone 709,116) were purchased from R&D Systems.

### Expansion of DNTs and lung cancer cell lines

DNTs were expanded ex vivo from healthy donors as described previously [14]. In brief, blood samples were obtained from healthy donors upon consent with a protocol approved by the University Health Network (UHN) Research Ethics Board. DNTs were enriched by depleting CD4^+^ and CD8^+^ cells using RosetteSep™ human CD4- and CD8-depletion cocktails (Stemcell Technologies). The CD4 and CD8 depleted cells were cultured in 24-well plates pre-coated with 5 μg/ml anti-CD3 antibody (OKT3, eBioscience) for 3 days in RPMI-1640 (Thermo Fisher Scientific) supplemented with 10% FBS (Sigma) and 250 IU/ml IL-2 (Proleukin). Fresh IL-2 and OKT3 were added to the DNT cultures every 2–4 days. DNTs were harvested between day 15–20 and purity was assessed by flow cytometry prior to experiments. The mean purity of DNTs used in the study was ~ 94%.

The tumor cell lines H2279, H460, H125, A549, OCI-AML3 and Jurkat (E6–1) were obtained from ATCC. Primary NSCLC cell lines 12, 178, 426, 277, 655, 229, 239 and 137 were derived from NSCLC PDX models (Additional file 1: Table S1), which were established using a protocol approved by the UHN Research Ethics Board. Briefly, primary lines were established from single cell suspensions of their corresponding PDX grown in immune deficient mice [17, 18]. Mutation information of primary NSCLC cell lines was profiled by OncoCarta Panel v1.0 (Agena Bioscience, San Diego, CA). All cell lines were maintained in DMEM/F12 (Gibco) supplemented with 10% FBS and used at less than 15 passages in vitro.

### Cytotoxicity and blocking assays

1 × 10^6^ cells/ml NSCLC cell lines were labelled with 5 μM florescent Vybrant™ DiO in PBS (ThermoFisher Scientific) for 15 mins at 37 °C. After washing, the DiO-labelled targets were added to 96-well plates in 100 μl DMEM/F12 with 10% FBS at 1 × 10^5^ cells/ml. DNTs were added at different effector to target (E:T) ratios. After 14 h co-culture, non-adherent cells were collected and transferred to a new microtiter plate. Remaining adherent cells were dissociated with 0.25% trypsin-EDTA solution (Sigma) and collected. For non-adherent target cells, cells were collected at 4 h after co-culture. TO-PRO-3 (3 μM, ThermoFisher Scientific) was added to cell suspension to stain for dead cells and cells were analyzed by flow cytometry to determine the frequency of live and dead DiO^+^ target cells. The specific cytotoxicity of DNTs against NSCLC cell was calculated by: $\frac{\%{DiO}^{+} TO- PRO-{3^{+}}_{with\  DNT}-\%{DiO}^{+} TO- PRO-{3^{+}}_{with out\  DNT}}{100-\%{DiO}^{+} TO- PRO-{3^{+}}_{with out\  DNT}}\times 100.$ The E:T EC50 was calculated using a non-linear regression fit of all E:T ratios in Table 1. For IL-15 stimulated assays, DNTs were stimulated with or without 100 ng/ml IL-15 for 24 h, followed by coculturing with NSCLC cells in the presence or absence of 100 ng/ml IL-15 for another 14 h. In some cases, NSCLC cells were cultured with 100 ng/ml IL-15 or supernatants from DNTs stimulated with or without 100 ng/ml IL-15. The cytotoxicity of DNTs against NSCLC cells was determined by flow cytometry at 5:1 E:T ratio or as indicated, all culture conditions contained 250 IU/ml of IL-2.

**Table 1:** NSCLC cell lines have different susceptibilities towards ex vivo expanded DNTs

Cell line (Cell source)	% cytotoxicity (Mean ± SD) at different E:T ratios				E:T EC50
	20:01	10:01	5:01	2.5:01	
H2279 (ATCC)	97.22 ± 12.04	87.69 ± 11.65	47.69 ± 19.34	23.83 ± 13.53	4.03
H460 (ATCC)	89.27 ± 7.23	61.52 ± 8.30	30.09 ± 12.88	14.88 ± 8.75	4.86
12 (PDX)	83.43 ± 17.97	75.32 ± 18.10	50.66 ± 21.09	24.15 ± 10.97	5.76
178 (PDX)	83.03 ± 10.55	58.96 ± 12.30	34.43 ± 7.49	11.81 ± 2.83	6.7
426 (PDX)	83.00 ± 10.50	64.54 ± 13.74	40.79 ± 7.24	16.32 ± 4.46	7.47
277 (PDX)	81.25 ± 9.27	67.62 ± 14.95	47.25 ± 8.07	21.76 ± 6.73	7.56
655 (PDX)	81.14 ± 7.99	63.21 ± 8.85	31.84 ± 1.90	17.79 ± 1.77	7.97
229 (PDX)	80.64 ± 10.41	58.96 ± 10.73	27.98 ± 14.16	10.28 ± 5.43	8.86
H125 (ATCC)	78.62 ± 14.80	53.97 ± 19.28	24.05 ± 10.39	16.39 ± 8.94	9.53
239 (PDX)	63.57 ± 9.98	36.19 ± 16.83	11.82 ± 8.76	12.45 ± 3.81	16.8
A549 (ATCC)	60.12 ± 10.51	35.69 ± 5.41	18.28 ± 5.21	9.77 ± 4.50	16.93
137 (PDX)	53.66 ± 15.40	30.84 ± 13.30	16.67 ± 6.47	8.51 ± 2.90	20.57

Primary and established human NSCLC cell lines were cocultured with ex vivo expanded DNTs at various E:T ratios. The percentages of specific cytotoxicity against target cells were detected. E:T EC50 was calculated for each cell line

For blocking assays, blocking antibodies or isotype matched controls were cultured with DNTs for 1 h prior to co-incubation with target cells at E:T ratio = 5:1 for 14 h. For TCR and perforin and granzyme B inhibition, anti-TCR antibody min and washed away prior to co-incubation with target cells as previously described [14]. For mTRAIL and CMA inhibition assays, DNTs were cultured for 30 min in the presence of 100 nM concanamycin A (CMA) or DMSO prior to co-culture with cancer targets in the presence of anti-TRAIL antibody or isotype control with IL-2 or IL-2/IL-15 stimulation. For sTRAIL blocking, DNT conditioned supernatant was cultured with anti-TRAIL antibody for 4 h prior to addition of lung cancer cells for 14 h. Percent inhibition of cytotoxicity was calculated by measuring the change in cytotoxicity observed between co-cultures containing blocking antibody to respective isotype control, vehicle control (DMSO), or media.

### Elisa

DNTs were cultured in media containing IL-2 with or without IL-15 for 24 h, and cell-free supernatants were used to measure interferon gamma (IFNγ), tumor necrosis factor alpha (TNFα) and soluble TNF-related apoptosis-inducing ligand (sTRAIL) release using ELISA MAX kits (Biolegend) or Quantikine ELISA kits (R&D systems).

### Xenograft model

NOD.Cg-*Prkdc*^*scid*^ *Il2rg*^*tm1Wjl*^/SzJ (NSG) mice (Jackson Laboratories, Bar Harbor, ME) were maintained at the UHN animal facility. 6–8 week old male mice were subcutaneously inoculated with H460 cells or A549 cells (1 × 10^6^/mouse) on day 0. Three days later, mice were treated i.v. with PBS or DNTs (2 × 10^7^/mouse) on days 3 and 7 or on days 3, 7 and 10 in the presence of IL-2 or IL-2 plus IL-15. IL-2 alone or together with IL-15 was administered i.p. twice a week. Mice were sacrificed when the tumor diameter reached 2 cm. Tumor volume was calculated by length × width^2^ × 0.52.

### Statistical analysis

All graphs and statistical analyses were performed with GraphPad Prism 6. The data were analyzed by two-tailed Student’s *t* test, one-way ANOVA followed by Bonferroni’s post hoc test and two-way ANOVA followed by Bonferroni’s post hoc test. The results were expressed as mean ± SD. Statistical significance was set as *P* < 0.05.

## Results

### Expanded DNTs cells are innate T cells with a cytotoxic phenotype

**Fig. 1:**
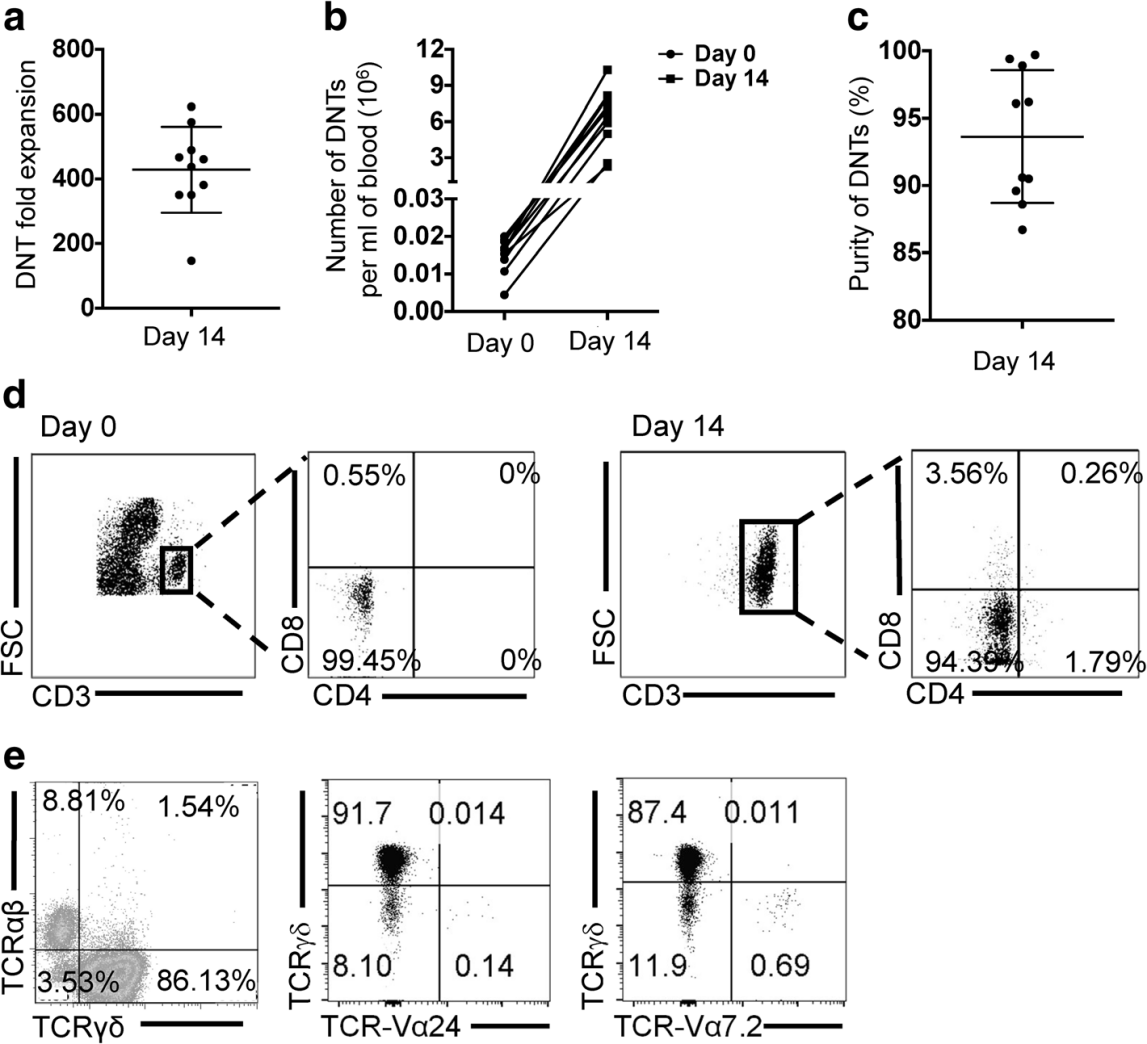
The subtypes of ex vivo expanded DNTs. a Fold expansion of DNTs on day 14 was calculated. **b** Numbers of DNTs expanded from 1 ml blood were calculated. **c, d** The purity of DNTs was detected on day 0 and day 14. **e** The percentages of αβ- and γδ-subtypes of DNTs were detected by flow cytometry. The subtypes of γδ-DNTs and αβ-DNTs were detected using Valpha24 (iNKT cell TCR) and Valpha7.2 (MAIT TCR)

To determine the potential of using ex vivo expanded human DNTs as an immunotherapy against solid tumors, we used our previously established protocol by which human DNTs can be expanded ex vivo from peripheral blood of healthy donors [14]. Using this protocol donor DNTs expanded 428.38 ± 133.17-fold in two weeks (Fig. 1a). Effectively, from 1 ml of blood, 15.18 ± 4.64 × 10^3^ DNTs on day 0 were expanded to 6.29 ± 2.49 × 10^6^ cells by day 14, with a purity of 93.63 ± 4.93% (Fig. 1b-d). As seen previously, the majority of expanded DNTs contained a mixture of αβ- (~ 10%) and γδ-T cells (> 80%), with minor populations (< 1%) of DNTs expressing known mucosal associated invariant T (MAIT) and invariant natural killer T (iNKT) cell receptors (Fig. 1e).

**Fig. 2:**
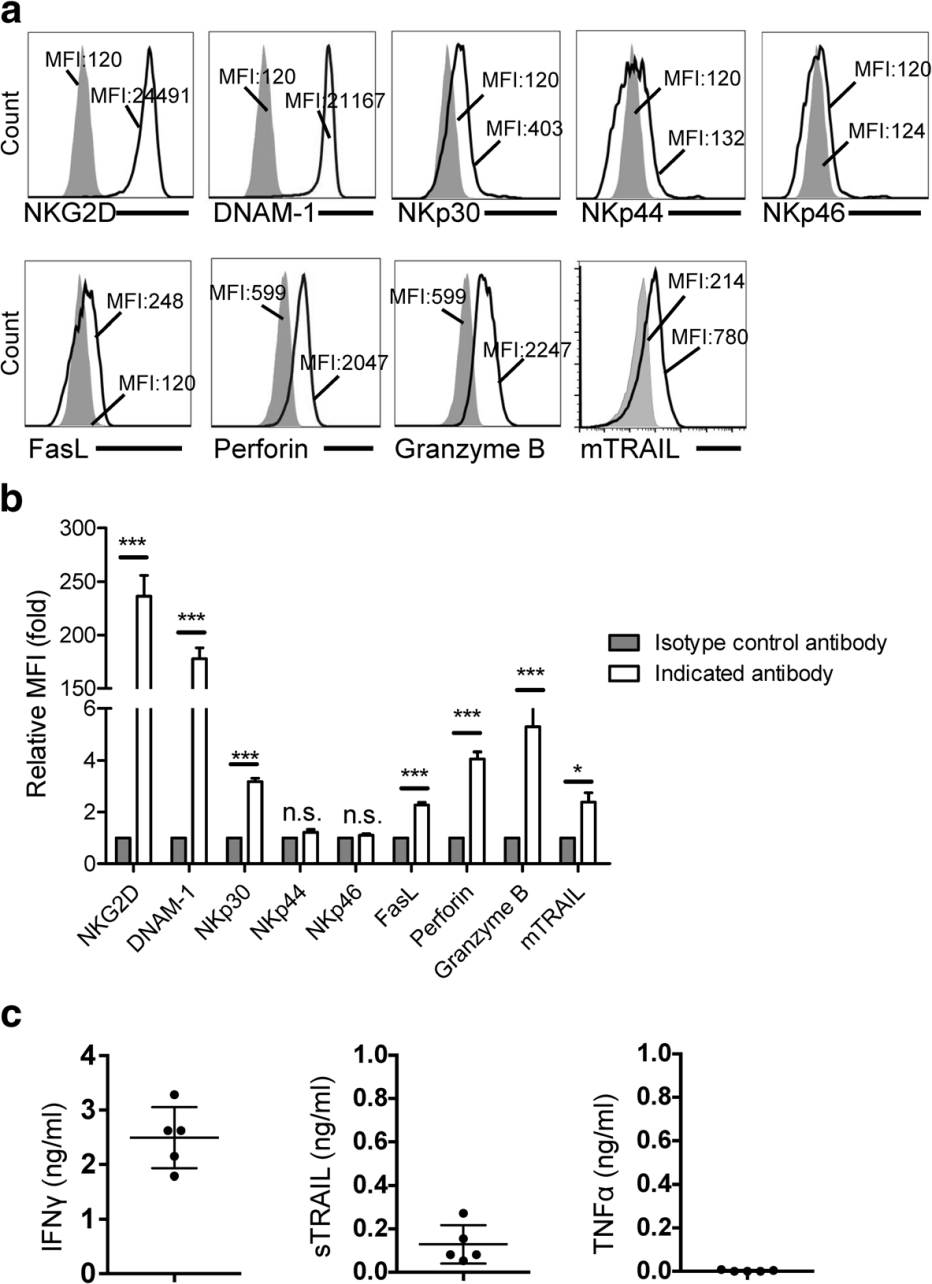
Activation molecules expressed on DNTs. a Ex vivo expanded DNTs were stained with indicated antibody (solid line) or isotype control antibody (filled histogram). **b** Relative MFI was calculated compared to DNTs stained with isotype control antibody. Data from 5 donors are shown. **c** DNT supernatants were collected, IFNγ, sTRAIL and TNFα were measured by ELISA. Each dot represents the data obtained from one healthy donor. *P < 0.05 and ***P < 0.001 compared

To further explore the potential of DNTs for use as effector cells against lung cancer, we screened DNTs for their expression of molecules known to be involved in immune cell mediated anti-tumor responses [19], including NKG2D, DNAM-1, the family of natural cytotoxicity receptors (NCR) NKp30, NKp44 and NKp46, FasL, membrane TRAIL (mTRAIL), perforin and granzyme B. Expanded DNTs showed a > 150-fold increase in MFI values for NKG2D and DNAM-1, and a 2-fold increase in NKp30, FasL, and mTRAIL expression compared to isotype controls (Fig. 2a and b). Expression of NKp44 and NKp46 was not detected. Expanded DNTs also expressed intracellular perforin and granzyme B (Fig. 2a and b) and secreted IFNγ and soluble TRAIL (sTRAIL), but not TNFα (Fig. 2c).

As a large proportion of DNTs are TCRγδ+, we screened for inhibitory killer cell immunoglobulin like receptor (KIR) expression, known to regulate γδ T cell clones [20]. Similar to the report by others [21], we found that KIRs are expressed clonally on DNTs, but that TCRαβ and TCRγδ subsets of DNTs expressed similarly high levels of cytotoxicity receptors, NKG2D and DNAM-1 (Additional file 1: Figure S1a and b). Given the similarity in cytotoxic cell surface marker expression between TCRαβ and TCRγδ subsets and their anti-leukemic function [14, 22], for the simplicity of future clinical application, the expanded DNTs were used in the following studies without further sorting.

### Ex vivo expanded DNTs effectively lyse human lung cancer cells in vitro and inhibit tumor growth in xenograft models

Recently we found that ex vivo expanded DNTs were cytotoxic towards human primary AML blasts and could reduce leukemia burden in PDX models of AML. Importantly, we demonstrated that DNTs were non-toxic towards normal cells and tissues [14]. To test the cytotoxic potential of DNTs against lung cancer, cells expanded from 8 healthy donors were cocultured with 8 primary and 4 established human NSCLC cell lines at varying E:T ratios (Table 1). Although cytotoxicity varied between different NSCLC lines, DNTs from all tested donors showed dose-dependent cytotoxicity towards both primary and established lung cancer cells (Table 1). The majority of NSCLC lines tested were highly susceptible to DNT-mediated lysis, with an E:T ratio EC50 of less than 10, such that an E:T ratio of 10:1 is capable of lysing 50% of NSCLC lines in cocultures. A549, and primary NSCLC lines 239, 137 were less susceptible, with a specific lysis E:T EC50 of greater than 16.

**Fig. 3:**
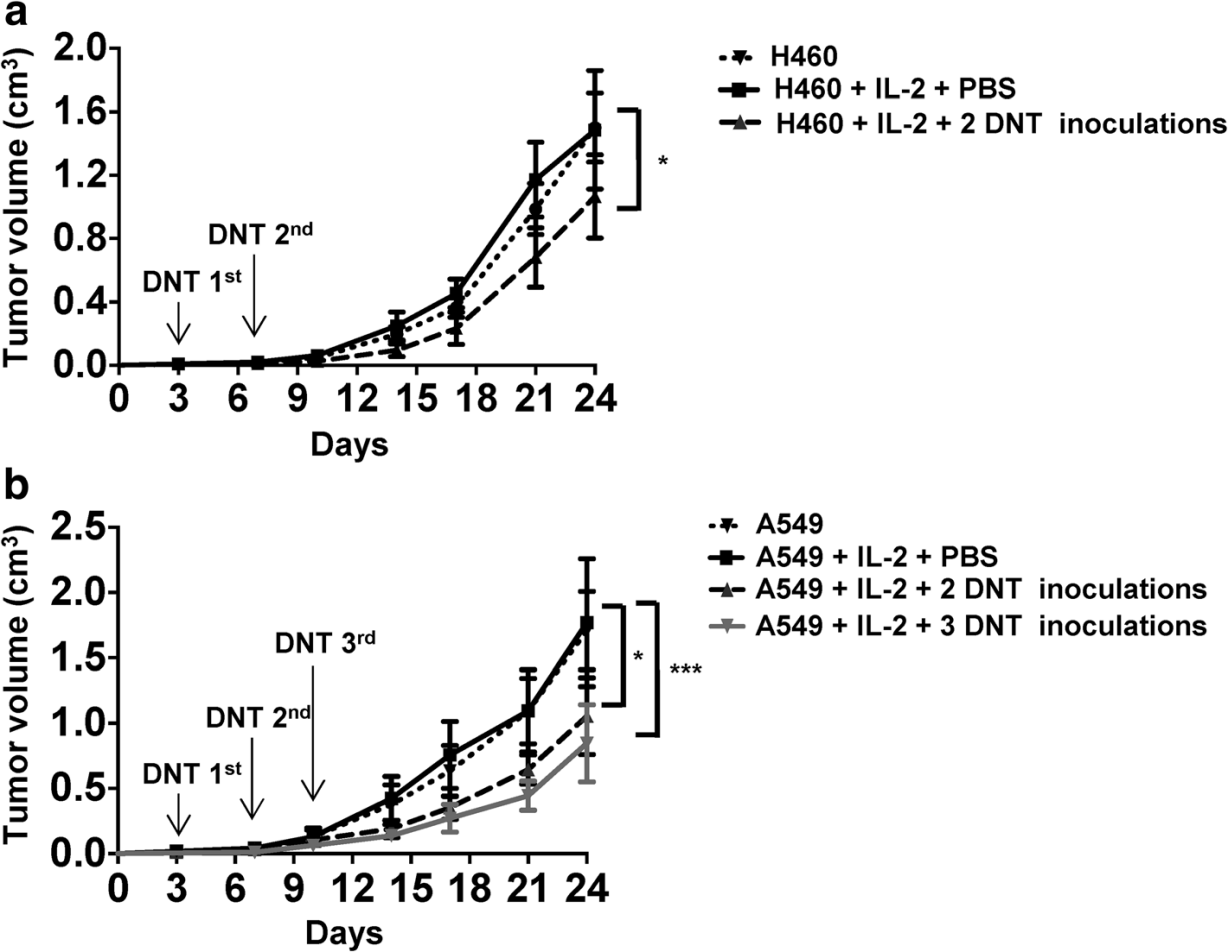
DNTs inhibit tumor growth in xenograft models. Mice bearing a H460 or **b** A549 xenografts were treated i.v. with PBS or DNTs (10^7^/injection) for 2 or 3 times in the presence of IL-2. Mice were sacrificed on day 24 (*n* = 5/group), tumor volumes were calculated. Arrows indicate the days of treatments. 2 injections of DNTs contained only 1st and 2nd DNT injections. Differences were calculated using two-way ANOVA followed by Bonferroni’s post hoc test. *P < 0.05 and ***P < 0.001 compared to H460 + PBS + IL-2 group in **a**, and A549 + PBS + IL-2 group in **b**. Data shown are representative of three independent experiments

To further determine the anti-tumor effect of DNTs in vivo, NSG mice were subcutaneously injected with H460 or A549 cells and divided into different treatment groups as shown in Fig. 3. Neither H460 nor A549 tumor growth was remarkably affected by IL-2 treatment alone. However, intravenous infusion of ex vivo expanded DNTs post-tumor inoculation resulted in a significant but modest reduction in tumor growth in both models. In mice that received H460 and 2 DNT treatments, tumor volume was reduced by 34.26 ± 17.81% on day 24 (Fig. 3a). Similarly, 2 and 3 DNT cell treatments resulted in 40.38% ± 14.83% and 51.05 ± 7.29% reduction in A549 tumor volume, respectively on day 24 (Fig. 3b). Compared to 2 injections of DNTs, 3 injections of DNTs led to a greater inhibition of tumor growth, therefore, 3 injections of DNTs were given in the following experiments. These data demonstrate that adoptive transfer of DNTs after tumor inoculation can inhibit lung cancer xenograft growth.

### DNTs utilize different mechanisms for cytolysis of NSCLC cells

**Fig. 4:**
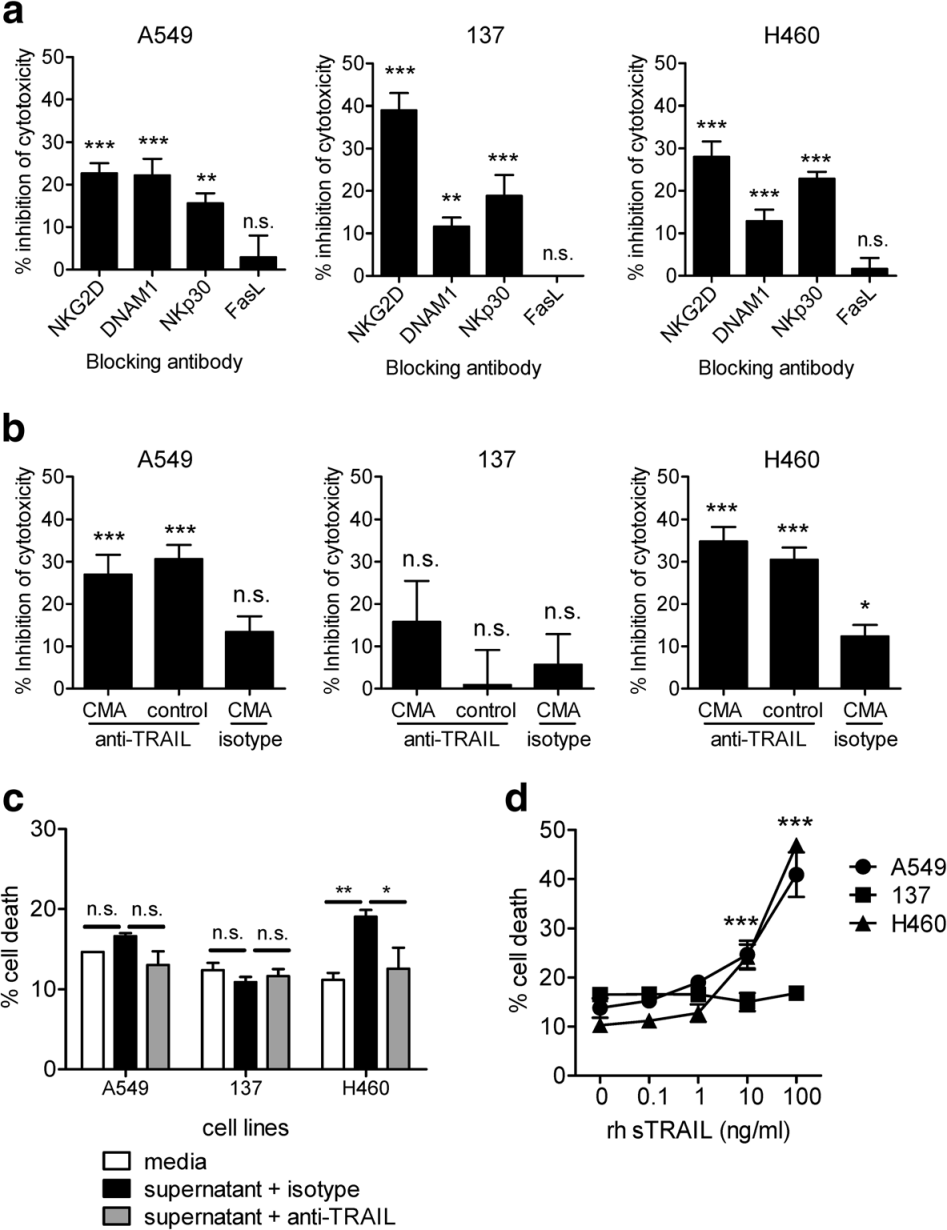
Cytotoxicity of DNTs against NSCLC cells can be mediated by different mechanisms. **a** The percentage of reduced target cell lysis by a specific antibody was compared to those in the presence of the corresponding isotype control antibody. **b** The percentage of reduced target cell lysis by CMA treatment with or without anti-TRAIL antibody was compared to those in the presence of vehicle DMSO and isotype controls. **c** NSCLC cell lines were cultured in media or IL-2 conditioned DNT culture supernatant in the presence of anti-TRAIL neutralizing antibody or isotype control. The death of NSCLC cells was determined by flow cytometry. **c** Different concentrations of rhsTRAIL were added to NSCLC cell cultures. The death of lung cancer cells was determined by flow cytometry. Statistical differences were calculated using one-way ANOVA followed by Bonferroni’s post hoc test. ns, not significant, *P < 0.05, **P < 0.01, ***P < 0.001. Data from one of three representative experiments (**a**) or cumulative of six independent experiments (**b**) are shown

To identify the molecules that are involved in the recognition and cytolysis of NSCLC cell lines, various blocking antibodies against the detected cell surface and soluble markers were used in cytotoxicity assays. Blocking of NKG2D, DNAM-1 and NKp30 resulted in significant inhibition of DNT-mediated cytolysis with different degrees of inhibition against different cell lines (Fig. 4a). Cytotoxicity towards cell line 137 showed the greatest dependency on NKG2D ligation compared to A549 and H460 cells as blocking this pathway resulted in a 39.04 ± 3.96% reduction in 137 cell cytolysis. Blocking the interaction of DNAM-1 with its ligands showed 22.18 ± 3.92% reduction in cytotoxicity towards A549 cells. In addition to innate receptor recognition of lung cancer, DNTs express TCRγδ which is known to respond to phosphoantigens. We found that while anti-TCRγδ antibody did alter Jurkat T cell cytolysis, TCR blockade did not alter lung cancer cell death (Additional file 1: Figure S2a). Further, in contrast to NKG2D, DNAM-1 and NCR markers, blocking of HLA had little effect on DNT-mediated cytotoxicity, consistent with the low expression of KIRs on DNTs (Additional file 1: Figure S2c).

Cytotoxic lymphocytes can utilize various mechanisms for cell mediated cytolysis [19]. We found that amongst the mechanisms analyzed, antibody blocking of TRAIL, led to reduced cytotoxicity towards A549 and H460 cells, but not 137 cells (Fig. 4b), whereas blocking of FasL and IFNγ had no significant effect on DNT-mediated lysis of three lung cancer cell lines analyzed (Fig. 4a and Additional file 1: Figure S3a). As perforin/granzyme B also have important roles in cytolysis, we determined whether CMA treatment of DNTs inhibited cytolysis in the absence of TRAIL. Whereas DNTs treated with CMA resulted in a significant inhibition of cytolysis against acute myeloid leukemia (> 80% inhibition; Additional file 1: Figure S3b), only a modest ~ 10% inhibition was observed against lung cancer, with statistical significance over control background observed only against H460 (Fig. 4b). Consistent with this observation, the combination of CMA treated DNT cells in the presence of anti-TRAIL antibody did not further reduce DNT cytotoxicity against A549 or modestly (~ 5%) reduced DNT cytotoxicity against H460 compared to anti-TRAIL alone (Fig. 4b). As TRAIL exists in membrane and soluble forms we next asked whether DNT derived sTRAIL may be involved. Whereas DNT supernatant, conditioned with only IL-2, induced cell death of H460, with trends towards cell death of A549, line 137 was not susceptible to DNT supernatant-mediated cell death (Fig. 4c). Further, to explore the role of sTRAIL, we found that addition of neutralizing anti-TRAIL antibody modestly but significantly reduced DNT supernatant-mediated cell death of H460, with trends towards reduction in A549 but not 137 (Fig. 4c). Interestingly, consistent with this finding, recombinant human sTRAIL induced a dose-dependent cell death of A549 and H460 cells, but not of cell line 137 (Fig. 4d). Taken together, these data indicate that the TRAIL pathway, and to a lesser extent perforin/granzyme B, are involved in DNT-mediated cytotoxicity in some but not all of the NSCLC cell lines tested.

### Lung cancer cell lines differ in ligand expression which track with mechanisms of DNT cytolysis

**Fig. 5:**
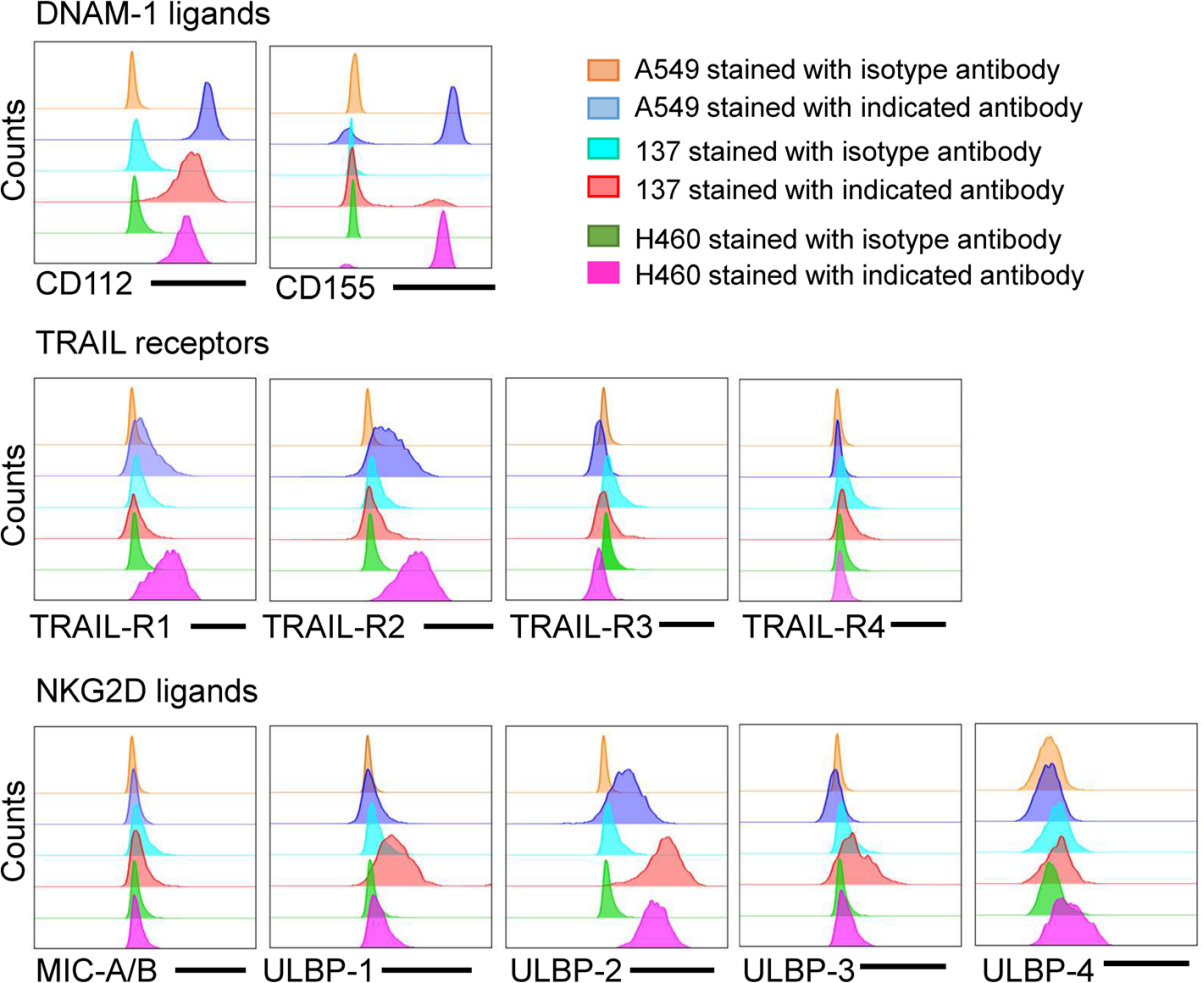
Ligand expression on NSCLC cell lines. Expression of NKG2D and DNAM-1 ligands, as well as, TRAIL receptors were detected on NSCLC cell lines by flow cytometry and compared to isotype controls for each line

With differences observed in the mechanisms of DNT-mediated cytolysis of different lung cancer cells, we hypothesized that the target cells may differ in their effector ligand expression. Indeed, different cancer cell lines showed differential expression for NKG2D and DNAM-1 ligands and TRAIL receptors. Whereas A549 and H460 cells showed increased expression of both DNAM-1 ligands, 137 cells only expressed CD112 and had a higher expression of NKG2D ligands, ULBP1 relative to A549 and H460 cells. Interestingly, TRAIL-R1 and TRAIL-R2 could only be detected on A549 and H460 cells, but not cell line 137 (Fig. 5), suggesting that DNTs may induce death of A549 and H460 cells via TRAIL receptors in addition to the NKG2D, DNAM-1 and NKp30 pathways. Collectively, these results demonstrate that DNTs can utilize different mechanisms to detect and lyse lung cancer cells.

### IL-15 enhances DNT-mediated anti-tumor activities in vitro and in vivo

**Fig. 6:**
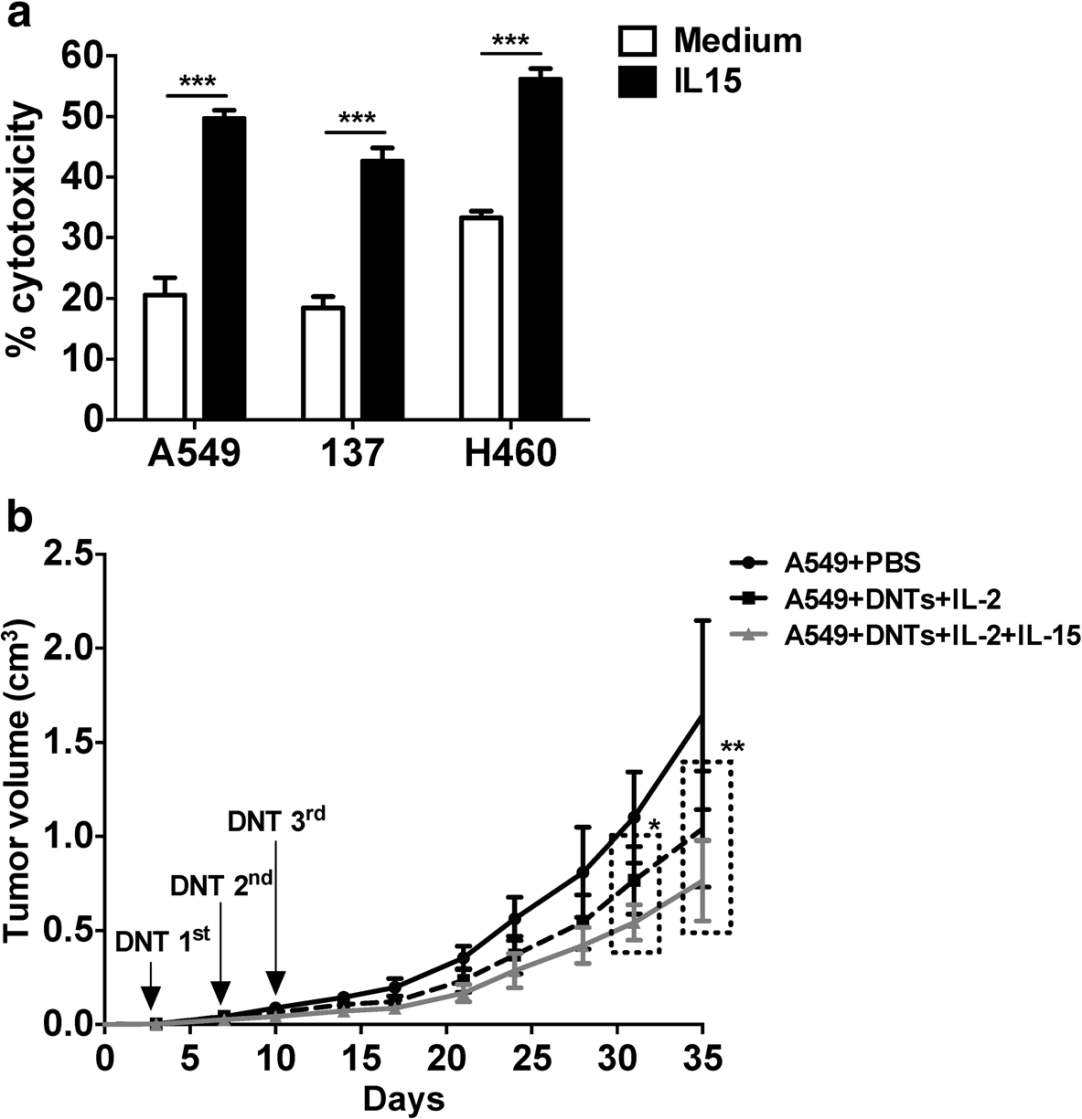
IL-15 enhances anti-tumor activity of DNTs in vitro and in vivo*.* **a** The cytotoxicity of DNTs against NSCLC cells in the presence or absence of IL-15 was determined by flow cytometry. This experiment was repeated using DNTs from 5 different donors with similar results. **b** A549 xenografts were treated with DNTs cultured with IL-2 or IL-2 plus IL-15, IL-2 and IL-15 were i.p. administered during the experiments. Mice were sacrificed on day 35 (*n* = 6/group). Two-tailed Student’s *t* test in **a**, two-way ANOVA followed by Bonferroni’s post hoc test in **b**. *P < 0.05, ***P < 0.001

Given that several NSCLC cell lines show some resistance to DNT-mediated cytolysis (Table 1), and lung cancer xenograft growth was modestly inhibited by DNTs, we determined whether IL-15, a well-known myeloid derived immune modulator [23], could augment DNT-mediated anti-tumor activity. DNTs were stimulated with rhIL-15 for 24 h prior to coculture with NSCLC cells. Compared to unstimulated DNTs, DNTs stimulated with rhIL-15 showed significantly increased cytotoxicity towards A549 (20.58 ± 1.60% vs. 49.71 ± 0.71%), line 137 (18.51 ± 1.04% vs. 42.66 ± 1.27), and H460 cells (33.27 ± 0.63% vs. 56.14 ± 1.01%, Fig. 6a).

To determine whether IL-15 could also enhance the anti-tumor effect of DNTs in vivo, NSG mice were inoculated s.c with A549 cells, followed by three i.v. infusions of DNTs cultured with IL-15 plus IL-2 or IL-2 only. Results showed a further 26.50 ± 22.68% reduction in tumor growth in mice injected with IL-15 plus IL-2-treated DNTs compared to IL-2-treated DNTs (Fig. 6b).

### IL-15 increases DNT-mediated cytotoxicity by upregulating their effector molecules

**Fig. 7:**
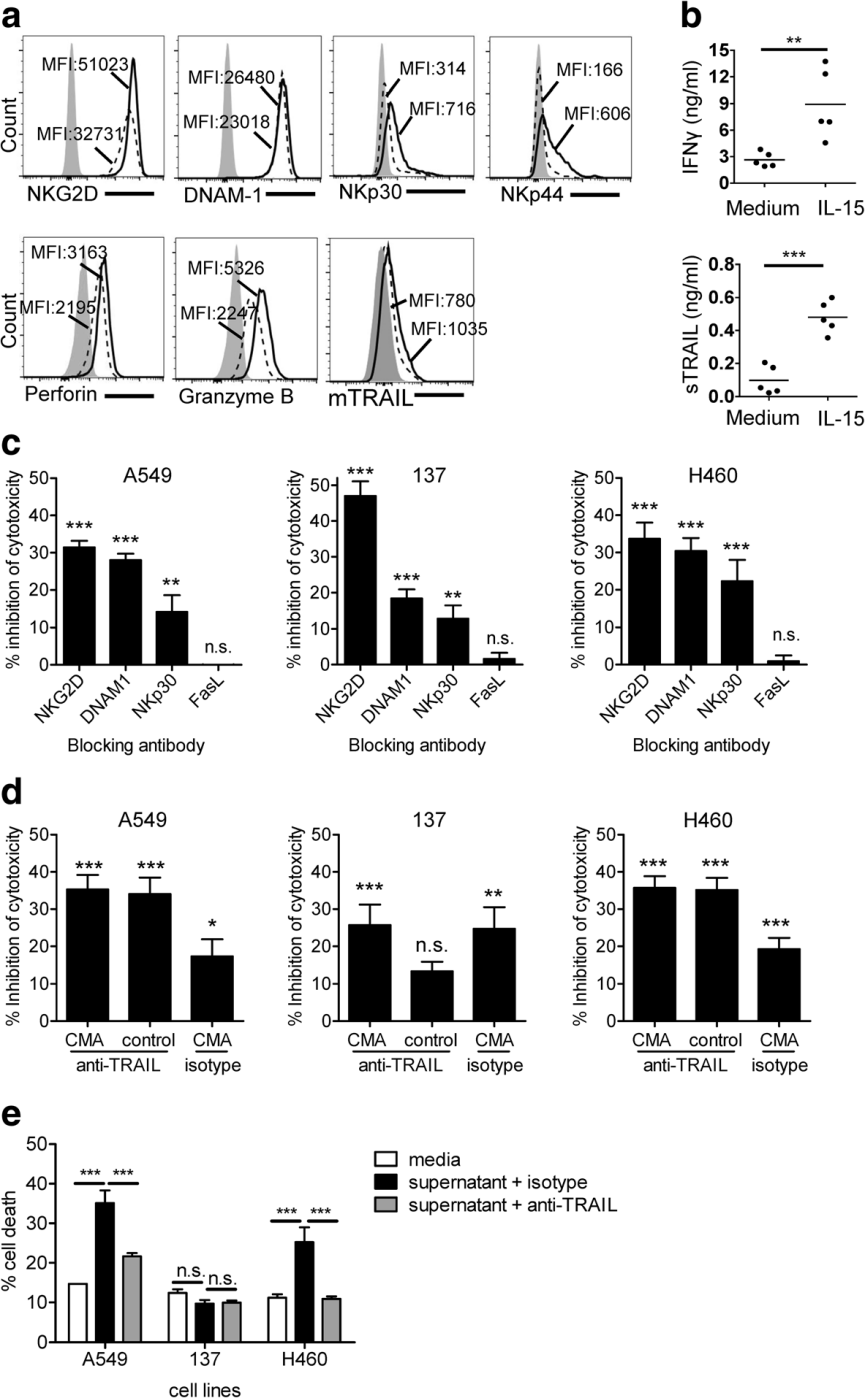
Cytotoxicity of DNTs is dependent on different mechanisms, which are augmented by IL-15. **a** Filled histograms represent isotype controls, dotted line histograms and solid line histograms represent DNTs in the absent and presence of IL-15, respectively. Numbers shown are the MFI values. **b** IFNγ and sTRAIL in the supernatant of DNTs stimulated with or without IL-15 were measured by ELISA. **c** The percentage of reduced target cell lysis by a specific antibody was compared to those in the presence of the corresponding isotype control antibody. **d** The percentage of reduced target cell lysis by CMA treatment with or without anti-TRAIL antibody was compared to those in the presence of vehicle DMSO and isotype controls. **e** NSCLC cell lines were cultured in the presence of media or IL-2/IL-15 conditioned DNT supernatant in the presence of neutralizing anti-TRAIL antibody or isotype control. The death of NSCLC cells was determined by flow cytometry. Statistical differences between different treatment groups were calculated using Two-tailed Student’s *t* test, for **b**. One-way ANOVA followed by Bonferroni’s post hoc test in **c**, **d** and **e**. ns, no significant, **P < 0.01, ***P < 0.001. Data from one of two or three representative experiments (**b**, **c**, **e**) or cumulative of six independent experiments (**d**) are shown

To understand how IL-15 augments DNT-mediated cytotoxicity, we first determined surface marker expression on DNTs following IL-15 stimulation. IL-15 treatment of DNTs upregulated early activation markers CD69 and CD25 (Additional file 1: Figure S4a). Importantly, IL-15 enhanced expression of cell-surface markers NKG2D, NKp30 and induced expression of NKp44 on DNTs (Fig. 7a). No changes were observed in FasL and NKp46 expression (Additional file 1: Figure S4b). Furthermore, addition of IL-15 had a modest effect on mTRAIL expression (Fig. 7a) and no change in TNFα production (Additional file 1: Figure S4c) but significantly increased secretion of IFNγ and sTRAIL by DNTs (Fig. 7b).

To determine if upregulation of specific effector molecules by IL-15 may alter previously detected mechanisms of lung cancer cytolysis, blocking studies were performed. Indeed, the cytotoxicity-mediated by IL-15 stimulated DNTs was significantly reduced to the levels of unstimulated DNTs (Fig. 4a) when NKG2D, DNAM-1 and NKp30 was blocked (Fig. 7c), suggesting a similar mechanism of recognition against NSCLC cell lines by IL-15-stimulated and non-stimulated DNTs. Again, line 137 showed a greater dependence on NKG2D and less dependence on DNAM-1 and NKp30 ligation (Fig. 7c). Interestingly, even though NKp44 was upregulated by IL-15, DNTs did not utilize this receptor in cytolysis of the three lung cancer cell lines tested (Additional file 1: Figure S5a).

Differing from non-stimulated DNTs, IL-15 treatment lead to some dependency on perforin/granzyme B pathway for cytolysis as a statistically significant inhibition against all lines tested was observed after CMA treatment (Fig. 7d). As line 137 showed the most resistant phenotype against DNT-mediated cytolysis, but was resistant to TRAIL-mediated cell death, CMA treatment significantly inhibited line 137 cytolysis even in the presence of anti-TRAIL antibody (Fig. 7d). Additionally, consistent with the pattern of sTRAIL on cancer cell death, supernatants from IL-15 stimulated DNT cultures induced significant toxicity to both A549 and H460 cells, but not to cell line 137 (Fig. 7e). Taken together, these data support the notion that DNTs can target lung cancer cells via different mechanisms that can be augmented by IL-15, and the expression levels of ligands and receptors on cancer cells dictate DNT cell mode of action.

## Discussion

DNT cell therapy emerges as a promising adoptive immunotherapy for cancer treatment. Recent data demonstrate that DNTs are able to target a broad range of leukemic cells in a non-donor restricted fashion [14, 16], and infusion of DNTs expanded from healthy volunteers significantly reduced leukemia load in AML PDX models without any observed toxicity [14]. Consistent with the anti-tumor nature of DNTs, we found that DNTs derived from all 8 donors tested showed cytotoxicity towards a panel of 12 NSCLC cell lines, demonstrating consistency across different healthy donors and further supporting the notion that DNTs target cancer cells in a donor-unrestricted manner (Table 1). DNT treatment of mice after tumor inoculation resulted in a significant but moderate inhibition of tumor growth when compared to untreated mice (Fig. 2). Though moderate, the reduction in tumor growth, given similar cellular doses, was consistent with that observed in pre-clinical models of CIK for NSCLC in the absence of additional interventions [24–26], but differ from the ability of CAR-T cells to reduce established tumor growth [12, 27].

A large proportion of expanded DNTs are γδ T cells, given the cytotoxic nature of γδ T cells, many phase I trials pursuing γδ T cell therapy have been reported in renal, prostate, breast, and lung cancers [28, 29]. Most studies used phosphoantigens to expand patient peripheral blood γδ T cells. All studies indicate that the adoptive transfer of ex vivo expanded γδ T cells is a well-tolerated therapy but with limited efficacy [28]. This may be due to the method of purification and expansion of γδ T cells as phosphoantigens selectively expand Vγ9δ2 T cells [28]. Given the heterogenic nature of solid tumors, selecting for particular clones or subsets may limit overall efficacy of anti-tumor responses. Furthermore, though γδ T cells have been reported to have adverse roles in cancer, these may be limited to tumor resident subsets [30, 31]. Our expansion protocol starts with depleting CD4^+^ and CD8^+^ T cells followed by polyclonal expansion of the remaining T cells with anti-CD3 antibody, which results in DNTs with a mixture of different subsets of γδ- as well as αβ-DNTs that are highly cytotoxic to tumor cells. Unlike CAR-T therapies or TCR-restricted therapies, both γδ T cells and DNT therapy do not rely on a priori knowledge of tumor specific antigens and require no genetic modification, but like CAR-T and CIK therapy may rely on increased trafficking and persistence or inhibition of the tumor microenvironment to improve clinical efficacy [27].

Additionally, DNTs expanded from healthy volunteers under good manufacturing practice (GMP) conditions can be cryopreserved with long shelf life and reserved function in vitro and in vivo [16]. Importantly, infusion of allogenic DNTs does not cause graft-vs.-host disease nor a host-vs.-graft reaction [16]. Collectively, these features allow DNTs to be developed as an “off-the-shelf” cellular therapy which has been approved for first-in-human clinical trial to treat high-risk AML patients (NCT03027102). The results of the trial and this study will support the initiation of a phase I clinical trial using DNTs to treat lung cancer patients.

Expanded DNTs expressed markers consistent with a cytotoxic phenotype, including expression of NKG2D, DNAM-1, and NKp30 as well as expression of intracellular granzyme B and perforin and secretion of IFNγ (Fig. 2). Furthermore, this phenotype was consistent amongst both TCRαβ and TCRγδ DNT subsets. While activated natural killer (NK) cells [32], γδ-T cells [33], and plasmacytoid dendritic cells [34, 35] show expression of NKp44 and NKp46, only NKp44 was upregulated on expanded DNTs with addition of IL-15. In delineating the mechanisms involved in DNT-mediated anti-tumor activities, we found that blockade of NKG2D, DNAM-1 and TRAIL differentially reduced the ability of DNTs to kill different lung cancer cells (Fig. 4a). In addition, we found that blocking of NKp30 also inhibited DNT-mediated cytotoxicity against lung cancer cells although to a lesser extent compared to the contributions of NKG2D and DNAM-1 pathways. The Fas/FasL pathway plays an important role in lymphocyte-mediated apoptosis under certain circumstances [19]. DNTs express a low level of FasL, which was not critical for DNT-mediated cytotoxicity against NSCLC cells (Fig. 4a).

A noticeable heterogeneity in the susceptibility to DNT cytolysis was observed among the 12 lung cancer cell lines tested (Table 1). This was not dependent on tumor subtype as the primary lung cancer cell line panel was derived from adenocarcinoma (Additional file 1: Table S1), with the exception of H460 (large-cell carcinoma) and H125 (adenosquamous carcinoma). Rather, differences in susceptibility were dependent on the expression of ligands on tumor cells that can be recognized by DNTs. Using tumors with varying levels of susceptibility, we found that all tumors showed some dependency on surface recognition of NKG2D, DNAM-1, and to a lesser extent NKp30 ligands. The level of expression of these ligands seemed to track with DNT mediated cytolysis of individual lines, for example, NKG2D blockade was highly effective in blocking cytolysis against 137, a cell that express higher NKG2D ligands relative to others. Similarly, DNAM-1 blockade did not affect 137 cytotoxicity as much as other cell lines, potentially due to reduced expression of DNAM-1 ligand CD155. Whereas DNT-mediated cytotoxicity to leukemic cells was largely dependent on IFNγ and perforin/granzyme B [14], this was not the case for lung cancer as blocking these using similar protocols only modestly affected DNT-mediated cytolysis (Additional file 1: Figure S5b).

We found that DNTs produced sTRAIL and the production was further increased when stimulated with IL-15 (Fig. 7b). Furthermore, addition of either recombinant sTRAIL or IL-15 conditioned DNT cell culture supernatant induced death of lung cancer cell lines that expressed TRAIL receptors (Fig. 7e), which could be blocked by anti-TRAIL neutralizing antibody (Fig. 7d, e). These data indicate that production of sTRAIL contributes to DNT cell-mediated anti-lung cancer activity. On the other hand, DNTs express a low level of mTRAIL (Fig. 7a) which may also contribute to cytolysis of lung cancer cells that express TRAIL receptors. Interestingly, as reported clinically, resistance to TRAIL mediated cytotoxicity is known in NSCLC tumors with several intrinsic defects at the receptor level and downstream signaling pathways described [36, 37]. We found that for line 137, a reduction in TRAIL receptor expression may explain resistance to DNT-mediated cytotoxicity. Given the importance of DNT recognition of lung cancer through NKG2D and DNAM-1 and as TRAIL blocking alone did not completely reduce DNT cytotoxicity in co-cultures, additional mechanisms not inhibited by Fas-FasL pathways such as perforin/granzyme B are likely involved. This was especially evident for DNTs treated with IL-15, which showed some dependency for perforin/granzyme B and was modestly but significantly inhibited by CMA treatment alone (Fig. 7d). Additionally, the combination of CMA with anti-TRAIL treatment revealed that in the absence of TRAIL, line 137 cytolysis was dependent on perforin/granzyme B. Surprisingly, for A549 and H460, the combination of anti-TRAIL with CMA treated DNTs did not further reduce cytotoxicity. This likely has to do with the modest role that CMA has on DNT-mediated cytolysis of these cell lines, but could also be due to inherent flaws of blocking studies that rely on reagents which may not completely inhibit intended targets. Nonetheless, these assays provide mechanistic insights not otherwise described. With the heterogeneity of NSCLC, our data suggest that DNT-mediated recognition and cytolysis of NSCLC is dependent on both expression of cancer associated ligands and the status of TRAIL resistance. An understanding of these expression patterns will help guide patient selection that may be responsive to adoptive DNT treatment.

IL-15 has been reported as tolerable for use in patients with metastatic melanoma or metastatic renal cell carcinoma, with patients showing altered homeostasis of NK cells, γδ T cells and CD8^+^ T cells in peripheral blood after treatment [38]. Similar to its ability to enhance the anti-tumor effect of NK cells and γδ T cells [39, 40], IL-15 also enhanced the anti-tumor effect of DNTs against NSCLC both in vitro and in vivo (Fig. 6)*.* Interestingly, IL-15 augmented DNT function by increasing the expression of effector molecules on DNTs (Fig. 7), potentially reducing the activation threshold required for the anti-tumor activity of DNTs. Though DNTs express NKp44 after IL-15 stimulation, NKp44 was not involved in IL-15-mediated lysis against NSCLC. Studies have reported that IL-15 can up-regulate NKG2D, DNAM-1 and TRAIL expression in NK cells and enhance their cytolysis against various tumors [40]. Consistently, IL-15 stimulated DNTs were more cytolytic towards NSCLC due to the upregulation of activation receptors by DNTs.

Taken together, these findings suggest that DNTs possess a “toolbox” which includes various effector molecules. Depending on the type of cancer targets and their expression of ligands/receptors, DNTs can utilize different tools to target different cancer cells. This feature allows DNTs to target a wide range of cancer cells including primary myeloid leukemia cells obtained from a large panel of patients [14] and various types of NSCLC cells (Table 1). Collectively, these results show that even in the context of tumor heterogeneity, DNTs may be poised with anti-tumor ability and share a similar dependency on cytotoxic markers as NK cells and CD8^+^ T cells [32, 41–43].

## Conclusions

Our study demonstrated that ex vivo expanded DNTs are effective at targeting a large array of NSCLC cell lines in vitro and moderately inhibiting lung cancer growth in vivo. The anti-tumor effect of DNTs is achieved by utilizing various mechanisms that depend on the presence of tumor ligands, and those mechanisms can be enhanced by the addition of IL-15. These data indicate that DNTs represent a promising new approach for treatment of lung cancer either alone or in combination with IL-15.

## Additional file

Additional file 1: **Figure S1.** DNT expression of inhibitory KIR and subset cytotoxicity markers. **Figure S2.** Effect of blocking TCR and HLA on DNT-mediated lysis of lung cancer cells. **Figure S3.** Mechanisms of DNT mediated cytotoxicity of lung cancer and AML3. **Figure S4.** IL-15 activates DNTs but has no effect on some effector molecules. **Figure S5.** The cytotoxicity of DNTs against NSCLC cells is not dependent on some effector molecules. **Table S1.** Histological classification and common mutation found within primary established NSCLC cell lines. (PDF 742 kb)

## Abbreviations

AML: acute myeloid leukemia
CAR-T cell: chimeric antigen receptor T cell
CIK: cytokine-induced killer cells
CMA: concanamycin A
DNTs: double negative T cells
E:T: effector to target
FasL: Fas ligand
GMP: good manufacturing practice
IFNγ: interferon gamma
iNKT: Invariant natural killer T cell
KIRs: killer cell immunoglobulin like receptors
MAGE-A3: melanoma-associated antigen-A3
mTRAIL: membrane TNF-related apoptosis-inducing ligand
NCR: natural cytotoxicity receptors
NK cells: natural killer cells
NSCLC: non-small cell lung cancer
NSG: NOD.Cg-*Prkdc*^*scid*^ *Il2rg*^*tm1Wjl*^/SzJ
PDX: patient-derived xenograft
sTRAIL: soluble TNF-related apoptosis-inducing ligand
TNFα: tumor necrosis factor alpha
UHN: University Health Network
