# Supplementary material for: Human double negative T cells target lung cancer via ligand-dependent mechanisms that can be enhanced by IL-15
Source: J Immunother Cancer. 2019 Jan 22;7:17. doi: 10.1186/s40425-019-0507-2 (PMC6343266; doi:10.1186/s40425-019-0507-2)
Supplement: Supplementary file 1 — Figure S1. DNT expression of inhibitory KIR and subset cytotoxicity markers. Figure S2. Effect of blocking TCR and HLA on DNT-mediated lysis of lung cancer cells. Figure S3. Mechanisms of DNT mediated cytotoxicity of lung cancer and AML3. Figure S4. IL-15 activates DNTs but has no effect on some effector molecules. Figure S5. The cytotoxicity of DNTs against NSCLC cells is not dependent on some effector molecules. Table S1. Histological classification and common mutation found within primary established NSCLC cell lines. (PDF 742 kb) [file 40425_2019_507_MOESM1_ESM.pdf]

Supplementary Figure S1

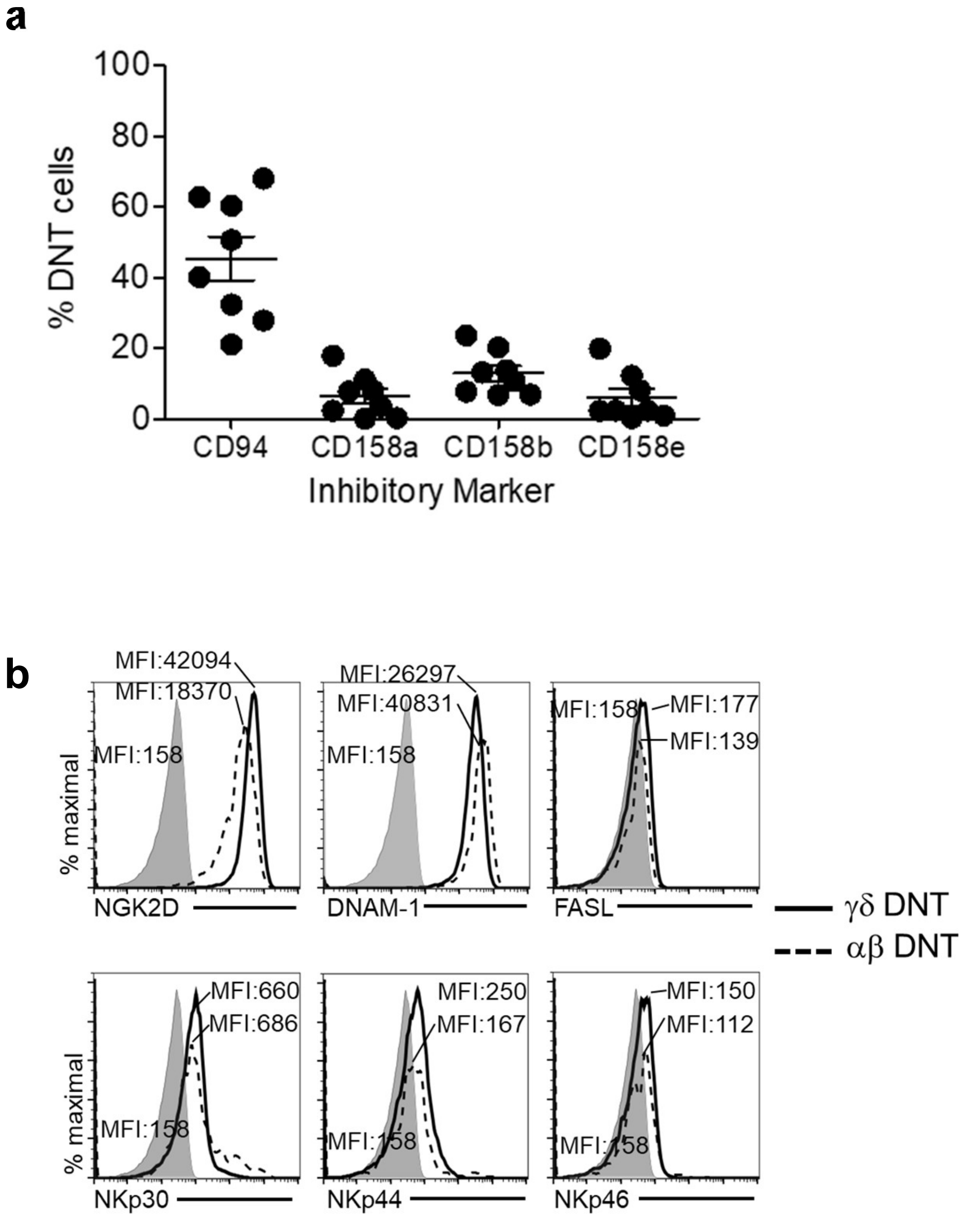

Supplementary Figure S1. DNT expression of inhibitory KIR and subset cytotoxicity markers. a. DNT expression of CD94 and KIR receptors on eight donors. Dot represents individual donor and % expression of molecule amongst DNT cells. **b.** DNT subset analysis of cytotoxicity receptors. Filled histograms represent isotype controls, dotted lines histograms represent αβ-DNTs and solid lines histograms represent γδ-DNTs. Numbers shown are the MFI values..

Supplementary Figure S2

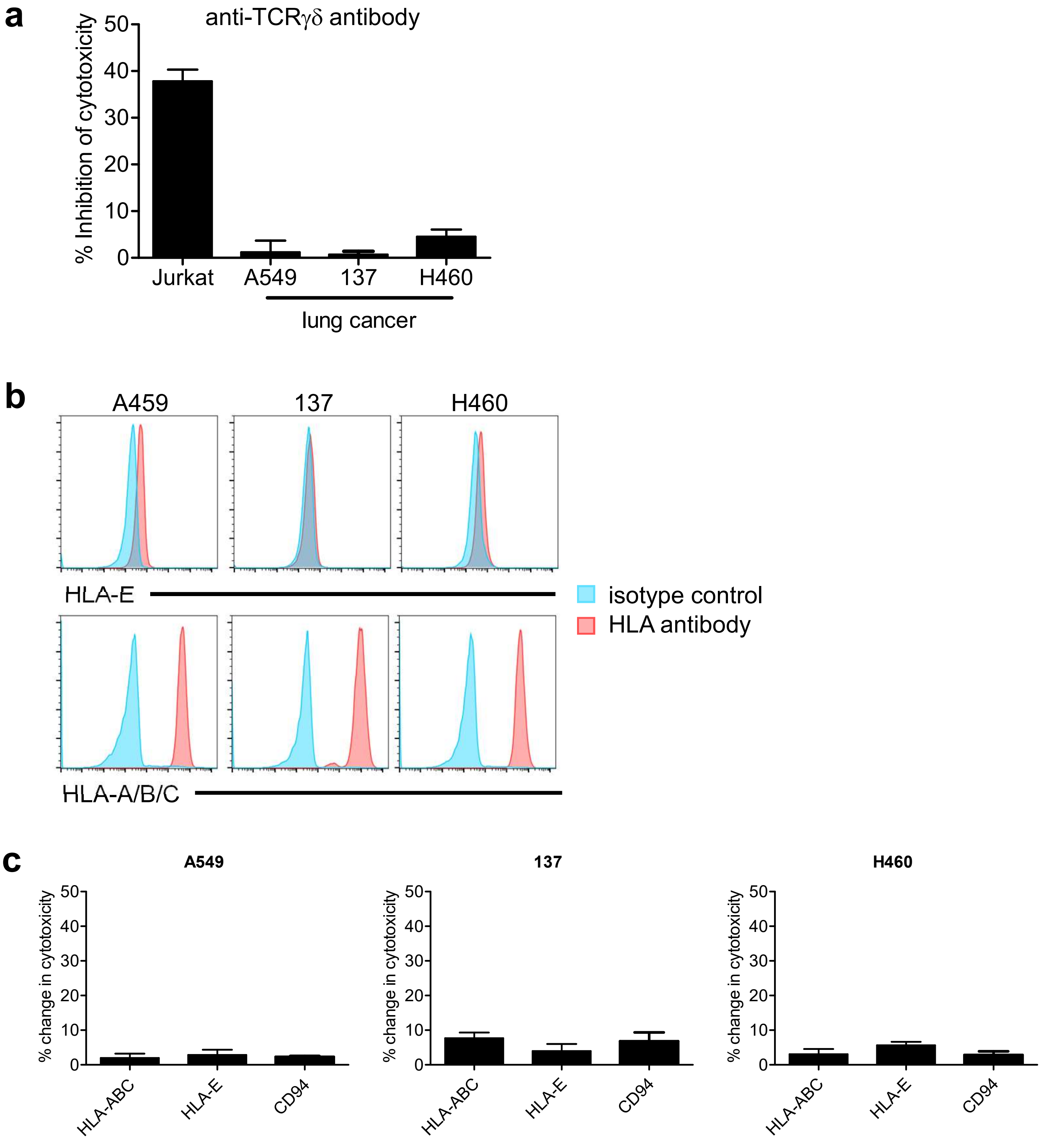

Supplementary Figure S2. Effect of blocking TCR and HLA on DNT-mediated lysis of lung cancer cells. A. Anti-TCRγδ antibody was used to block DNT-mediated cytolysis towards various cancer cell lines. **B.** Expression of HLA molecules known to interact with KIRs on lung cancer cells. **C.** Anti-HLA antibodies and anti-CD94 on DNT-mediated cytolysis of lung cancer cells.

Supplementary Figure S3

a

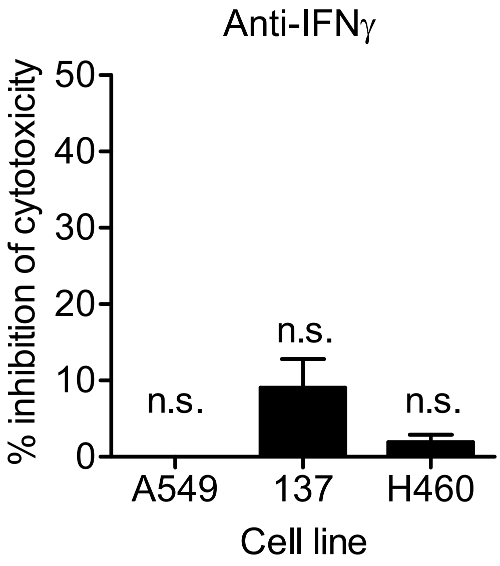

b

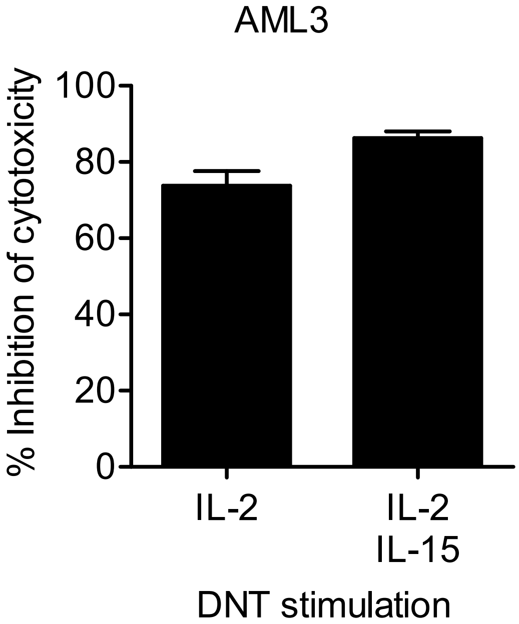

Mechanisms of DNT mediated cytotoxicity of lung cancer and AML3. a. DNTs were cocultured with NSCLC cell lines in the presence of anti-IFNγ antibody. % inhibition calculated relative to isotype control. **b.** DNT were treated with CMA then cocultured with acute myeloid leukemia cell line AML3. % inhibition calculated relative to DMSO vehicle treated cells.

Supplementary Figure S4

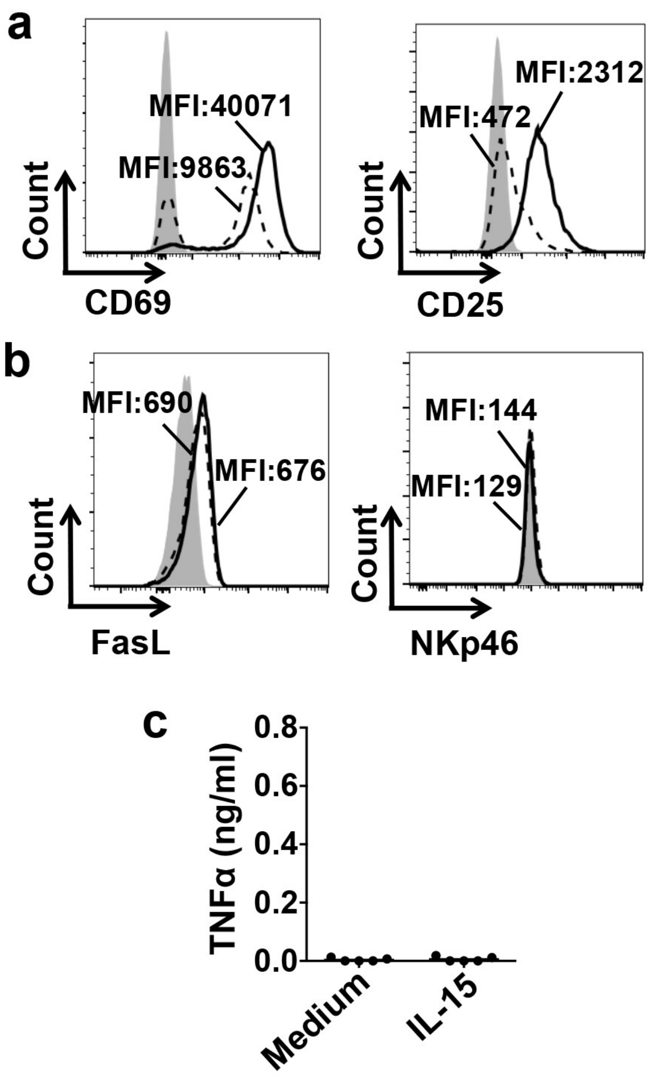

IL-15 activates DNTs but has no effect on some effector molecules. a, b Filled histograms represent isotype controls, dotted line histograms represent DNTs in the absent of IL-15 and solid line histograms represent DNTs in the present of IL-15. **c** TNFα in the supernatant of DNTs stimulated with IL-2 in the presence of absence of IL-15 was measured by ELISA.

Supplementary Figure S5

a

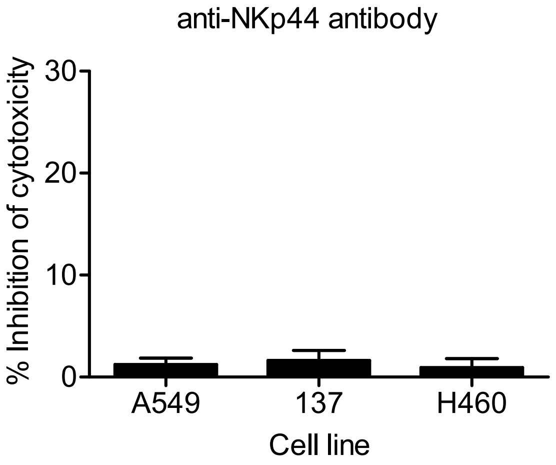

b

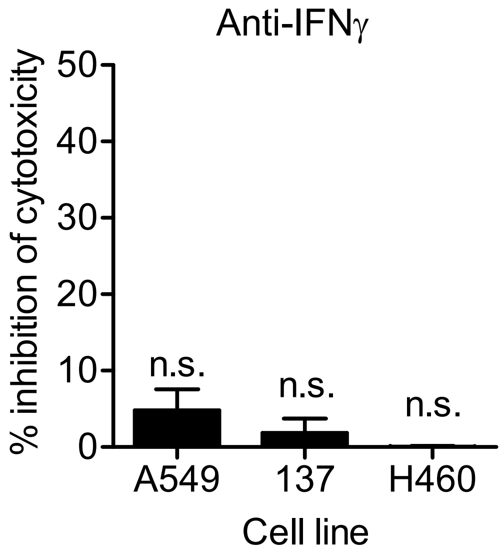

The cytotoxicity of DNTs against NSCLC cells is not dependent on some effector molecules. Anti-NKp44 (**a**) or anti-IFNγ (**b**) antibody was added to DNTs and NSCLC cells co-cultures. The percent inhibition of cytotoxicity was calculated relative to isotype control.

Supplementary Table S1

Histological classification and common mutation found within primary established NSCLC cell lines.

Primary cell line ID	Histology	Driver mutation*	Patient (Male/Female)
12	adenocarcinoma (ADC)	*BRAF*	F
178	adenocarcinoma (ADC)	no mutation	F
426	adenocarcinoma (ADC)	not tested	F
277	adenocarcinoma (ADC)	*EGFR*	M
655	adenocarcinoma (ADC)	no mutation	M
229	adenocarcinoma (ADC)	no mutation	M
239	adenocarcinoma (ADC)	no mutation	M
137	adenocarcinoma (ADC)	*EGFR*	F
